# Accelerating Discontinuous Precipitation to Increase Strength by Pre-Deformation in Cu-Ni-Si Alloys

**DOI:** 10.3390/ma17225658

**Published:** 2024-11-20

**Authors:** Yicheng Cao, Wei Luo, Wenjing Zhang, Haofeng Xie, Zhen Yang, Zengde Li, Lijun Peng, Yunqing Zhu

**Affiliations:** 1 State Key Laboratory of Nonferrous Metals and Processes, GRINM Group Co., Ltd., Beijing 100088, China; caoyicheng1995@163.com (Y.C.); docmetal503@gmail.com (W.L.); lizengde@grinm.com (Z.L.); penglijun@grinm.com (L.P.); xz03080222@163.com (Y.Z.); 2 GRIMAT Engineering Institute Co., Ltd., Beijing 101407, China; 3 General Research Institute for Nonferrous Metals, Beijing 100088, China

**Keywords:** Cu-Ni-Si alloy, Ni_2_Si, pre-deformation, discontinuous precipitation, tensile strength, electrical conductivity

## Abstract

Discontinuous precipitation-strengthened Cu-Ni-Si alloys are highly regarded for their combination of high strength and excellent electrical conductivity. However, the slow process of discontinuous precipitation, typically requiring up to 24 h for complete formation, significantly increases the alloy’s production costs and limits potential improvements in its properties. This study addresses this issue by applying pre-deformation to Cu-6Ni-1.42Si alloys, which accelerated the discontinuous precipitation (DP) of Ni_2_Si by approximately 48 times, resulting in the formation of fast DP and full DP alloys. The fast DP alloy exhibited a smaller DP size and inter-distance than the full DP alloy, achieving a tensile strength of 1070 MPa and a conductivity of 38.5% IACS. In contrast, the full DP alloy had a slightly lower tensile strength (approximately 930 MPa) but a higher conductivity of 46% IACS. Both alloys outperform traditional Cu-Ni-Si alloys in strength while maintaining comparable conductivity. The accelerated DP technique improves mechanical properties without significantly sacrificing conductivity, offering a new approach for high-performance conductive materials.

## 1. Introduction

With the continuous advancement of technology, the demand for electronic and electrical devices is gradually increasing, particularly in the development of high-performance and high-quality equipment. This trend places greater performance requirements on the critical components within these devices, especially core elements such as lead frames and connectors, which directly impact the overall performance and reliability of the equipment. To meet the market’s expectations for high-performance devices, it is essential to enhance the strength and conductivity of these components. Among various materials, copper alloys are ideal choices for lead frames and connectors due to their excellent conductivity and mechanical strength. However, as performance demands become increasingly stringent, relying solely on the properties of traditional copper alloys is insufficient to satisfy the requirements of modern electronic devices. Therefore, it is necessary to explore methods to improve the strength of copper alloys while maintaining or enhancing their conductivity to achieve the comprehensive optimization of material performance. In this context, Cu-Ni-Si alloys have emerged as particularly notable due to their exceptional properties [1,2,3,4,5].

Cu-Ni-Si alloys play a significant role in electrical components due to their excellent strength and conductivity. With the continuous increase in industrial demand, researchers have devoted the past few decades to simultaneously enhancing the strength and conductivity of Cu-Ni-Si alloys to meet increasingly stringent performance standards [6,7,8,9,10,11,12,13,14,15,16,17]. In Cu-Ni-Si alloys, strength is primarily determined by the quantity, type, size, and uniform distribution of precipitates, while the conductivity is mainly influenced by the type and amount of soluble atoms dissolved in the copper matrix. Specifically, precipitates enhance the material’s strength by obstructing dislocation motion, whereas soluble atoms affect the electron transport pathways, thus altering conductivity. During the preparation of these alloys, a series of mechanical and thermal processing methods, such as solution treatment, aging treatment, and deformation, are typically employed. These processes not only effectively reduce the concentration of soluble atoms in the matrix, but also facilitate the formation of precipitates, introducing dislocations that interact with the precipitates for strengthening. This interaction is one of the key factors in improving both the strength and conductivity of the alloy. In particular, the type and quantity of precipitates formed during the aging process have a significant impact on the final properties of the alloy. For instance, different types of precipitates (such as continuous and discontinuous precipitates) can influence the mechanical and electrical properties of the material in distinct ways.

Nanometer-sized Ni_2_Si continuous precipitates (CPs) obtained through peak aging are uniformly distributed within the copper matrix. This structure can significantly enhance the strength of the alloy but often sacrifices its conductivity. This occurs because in the peak aging state, the soluble atoms do not precipitate in large quantities; the nickel and silicon atoms dissolved in the matrix induce lattice distortion, thereby reducing the material’s conductivity.

Conversely, lamellar discontinuous precipitates (DPs) formed through excessive aging can lead to a substantial precipitation of soluble atoms, enhancing conductivity. However, the relatively large size and spacing of DPs can adversely affect mechanical properties, potentially resulting in a reduction in strength. Nonetheless, recent studies by Han [18,19,20,21,22,23,24,25,26] and Semboshi [27,28,29,30,31] have indicated that under specific conditions, discontinuous precipitates can serve as an effective strengthening mechanism. Specifically, through the cold processing of Cu-Ni-Si and Cu-Ti alloys after aging, it is possible to achieve simultaneous improvements in both strength and conductivity. These studies indicate that following cold processing, the hard Ni_2_Si precipitates can be elongated and neatly aligned along the tensile direction, leading to a reduction in the spacing between discontinuous precipitates (DPs). This arrangement significantly enhances the strength of the alloy. Additionally, the coarsening of DPs can effectively purify the matrix, while the orderly alignment of DPs helps to reduce electron scattering, thereby improving conductivity. The combined effects of these two factors highlight the potential of Cu-Ni-Si alloys containing a substantial amount of DPs as novel materials with high strength and high conductivity.

However, to obtain a substantial amount of discontinuous precipitates in Cu-Ni-Si alloys, excessive aging treatment is necessary. This not only increases production costs, but also leads to the formation of coarsened second and third discontinuous phases at the grain boundaries. These coarsened phases do not undergo plastic deformation during the cold working of the alloy, thus failing to contribute to the alloy’s strength. Instead, they may serve as initiation points for crack propagation during plastic deformation, ultimately reducing the alloy’s ductility and negatively impacting its mechanical properties and workability. To mitigate manufacturing costs and avoid these adverse effects, researchers have begun to explore methods to accelerate the precipitation of discontinuous phases.

In the study of Cu-Ti alloys, some studies have proposed techniques such as multi-stage aging [32] and pre-deformation [33] to accelerate the precipitation of discontinuous phases (DPs). In previous research [34,35], it was found that introducing pre-deformation in Cu-Ni-Si alloys can similarly and significantly accelerate the generation of DPs, reducing the time required to achieve complete DPs from 1440 min to just 30 min. This improvement is attributed to the slip bands introduced during pre-deformation, which not only facilitate the nucleation of DPs, but also provide rapid atomic diffusion pathways. Slip bands are structural features that form in metallic materials under low deformation strain, with their formation mechanism being closely related to the crystallographic structure within the grains. When an external force is applied to the metal, dislocations within the crystal slip along specific slip planes, leading to localized deformation either on the material’s surface or internally, forming slip bands. These bands typically interact with grain boundaries without crossing them and are confined by the crystallographic structure of the metal [36,37]. Furthermore, some studies [38,39] have suggested that deformation bands, such as slip bands and shear bands, can act as channels for fast atomic diffusion, further enhancing material behavior during processes like pre-deformation. However, the specific effects of accelerated DP generation on alloy performance remain unclear and warrant further investigation to elucidate their potential mechanisms. In this study, we prepared two different Cu-Ni-Si alloys by performing aging treatments for varying durations following pre-deformation—one with fast DPs and the other with complete DPs. This research aims to explore the impact of accelerated discontinuous precipitation on the strength and conductivity of these two alloys after drawing, with the goal of providing new insights and methods for the optimization of Cu-Ni-Si alloys.

By effectively controlling the formation and distribution of precipitates, we anticipate laying a solid foundation for the development of key components in electronic and electrical devices. This not only contributes to enhancing the overall performance of the alloys, but also has the potential to promote their widespread application in high-performance settings.

## 2. Experiment Procedure

The Cu-6wt%Ni-1.42wt%Si alloy was prepared through induction melting, utilizing materials with purities of 99% copper, 99% nickel, and 99% silicon. The cast ingots were cut into rods with a diameter of 20 mm and were subjected to rotary forging, reducing the cross-sectional area by 75% to fracture the cast structure. Subsequently, a solution heat treatment was conducted at 980 °C for 1 h, followed by water quenching. The samples were then stretched through slot rolling to achieve an 18% reduction in area, introducing slip bands. This was followed by aging treatments at 500 °C for 30 min and 720 min to obtain fast discontinuous precipitates (DPs) and full discontinuous precipitates, respectively. Samples for fast DPs and full DPs underwent plastic deformation with area reductions ranging from 75% to 97.5%.

Vickers hardness testing was performed using a hardness tester (Model: HM-200, Mitutoyo Corporation, Kanagawa, Japan) with a load of 100 g and a dwell time of 15 s. Each sample was tested 10 times, and the average value was calculated. Conductivity was measured using a portable dual bridge resistivity meter (Model: Precision Double Bridge 2769, Yokogawa Electric Corporation, Tokyo, Japan) at an ambient temperature of 26 °C. Tensile tests on dog-bone and wire samples were conducted on an universal testing machine (Model: INSTRON 5982, Instron Corporation, Buckinghamshire, UK) with a strain rate of 1 mm/min and a gauge length of 25 mm.

For microstructural observation, equipment included an optical microscope (OM, Model: GX51, Olympus Corporation, Tokyo, Japan), a scanning electron microscope (SEM, Model: IT300, JEOL Company, Tokyo, Japan), a field emission scanning electron microscope (FESEM, Model: JSM7001F, JEOL Company, Tokyo, Japan), and a transmission electron microscope (TEM, Model: Tecnai G2 F20, FEI Company, Hillsboro, OR, USA). Each sample underwent cold embedding and mechanical polishing, followed by electrochemical etching in a 40% phosphoric acid solution, with etching times of 2 s for OM and SEM observations and 20–30 s for FESEM observations. TEM samples were thinned to 50 μm and then punched into 3 mm diameter disks, followed by dual-jet electro-polishing and argon ion milling for sample preparation.

## 3. Result and Discussion

**Figure 1:**
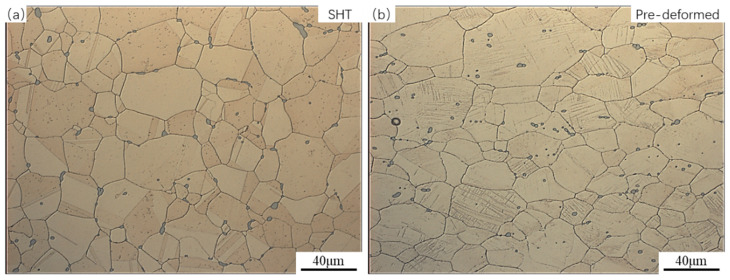
The optical microstructure of the Cu-6Ni-1.42Si alloy after solution treatment (**a**) and after pre-deformation (**b**).

Figure 1 shows the optical microstructure of the Cu-6Ni-1.42Si alloy after solution treatment (a) and after pre-deformation (b). From Figure 1a, it can be observed that the alloy exhibits equiaxed grains after solution treatment, with grain sizes ranging from 30 to 100 μm. Additionally, a large number of inclusions are distributed along the grain boundaries, which is attributed to the contents of Ni and Si in the alloy exceeding their maximum solubility in the Cu matrix, leading to an inability to dissolve. Previous studies by the authors [35] have found that inclusions can appropriately enhance the work hardening rate of the alloy, thereby increasing its strength. From Figure 1b, it can be noted that after an 18% area reduction due to cold deformation, the grains are slightly elongated, and some inclusions are distributed within the grains as the grain boundaries move, which may be beneficial for improving the alloy’s ductility. Within the grains, numerous slip bands can be observed; these slip bands can serve to provide nucleation sites for discontinuous precipitates and fast diffusion pathways for solute atoms during subsequent aging treatments, thus accelerating the generation and growth of DPs.

**Figure 2:**
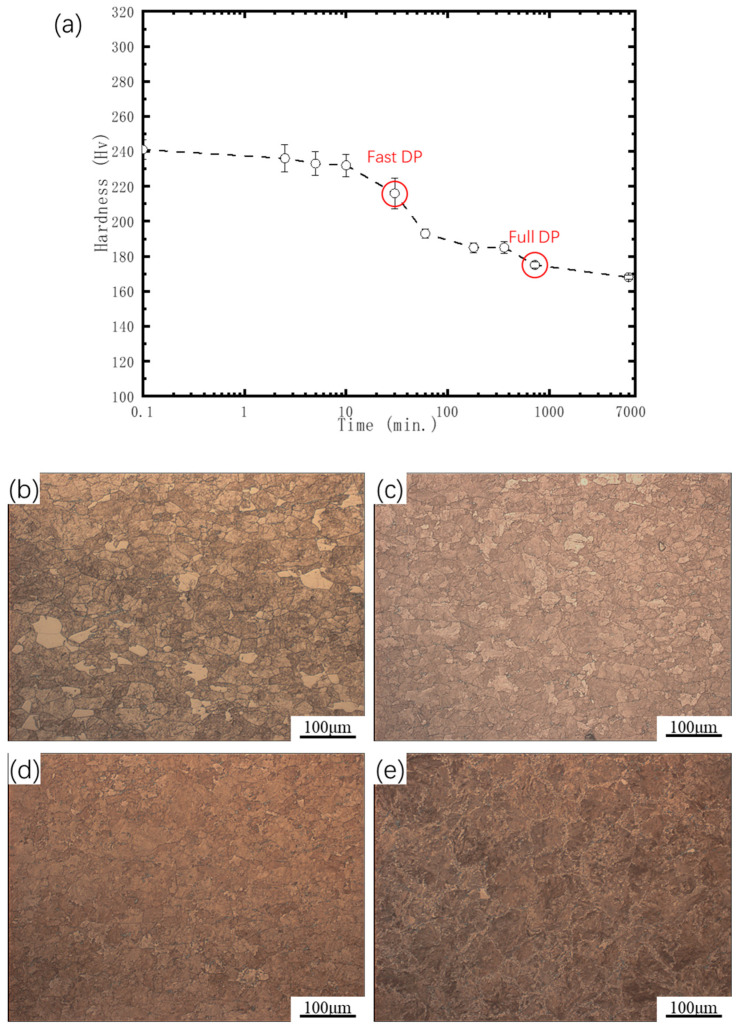
(**a**) Hardness change in pre-deformed Cu-Ni-Si alloys during aging at 500 °C for different times and microstructures of pre-deformed Cu-Ni-Si alloys aged for (**b**) 10, (**c**) 30, (**d**) 720, and (**e**) 6000 min.

Figure 2a shows the hardness variation in pre-deformed Cu-Ni-Si alloys aged at 500 °C for different durations. Notably, the hardness continuously decreases with increasing aging time, without exhibiting peak aging. This indicates that in the initial stage of aging, a significant amount of CP precipitation does not occur; instead, coarser DPs precipitate directly. After solution treatment, alloys that have not undergone pre-deformation typically exhibit a lower hardness. During the aging process, nano-sized CPs normally precipitate first, leading to an increase in hardness and the appearance of peak aging. Then, with extended aging time, larger DPs will precipitate, causing a reduction in hardness [35]. In this experiment, no peak aging was observed in the pre-deformed alloy, and the hardness began to decrease at the early stage of aging. Therefore, it is speculated that the alloy directly precipitated the DP phase without forming the CP phase. This behavior differs from that of alloys without pre-deformation, demonstrating that pre-deformation can accelerate the precipitation and growth of DPs. Figure 2b–e illustrate the metallographic structures of pre-deformed alloys aged at 500 °C for 10 min, 30 min, 720 min, and 6000 min, respectively. From the figures, it can be observed that a large number of DPs appear after only 10 min of aging, indicating that DPs generate directly in the pre-deformed alloy. At aging times of 30 min and 720 min, the area fraction of DPs was calculated using ImageJ software (version: 1.51j8, LOCI, University of Wisconsin, Madison, WI, USA), yielding 95.03% and 98.93%, respectively, demonstrating that the area fractions of the DP phases in the two samples are nearly identical. This indicates that the aging time required to achieve full DPs has been significantly reduced from the previously reported 24 h to only 30 min, accelerating the DP formation time by approximately 48 times.

**Figure 3:**
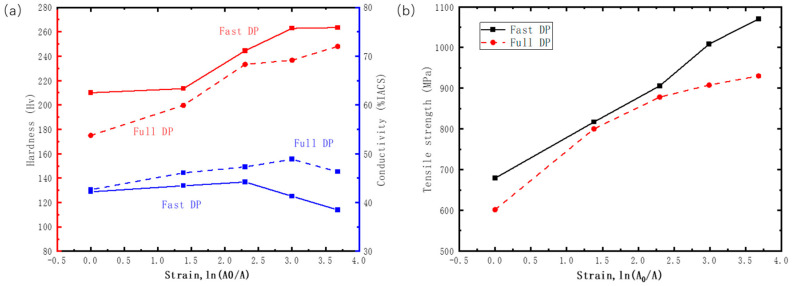
The variation of (**a**) hardness and electrical conductivity, and (**b**) tensile strength with strain in Cu-Ni-Si alloys with fast DPs and full DPs at room temperature.

Figure 3 shows the variations in hardness, conductivity (a), and tensile strength (b) after different levels of tensile strain. Following cold working, the hardness of both alloys increases with raising strain. The hardness of the samples with fast DPs is higher than that of the full DP samples at all levels of deformation. After aging for 30 min and 720 min, there is no significant difference in conductivity between the alloys. As the strain increases, the electrical conductivities of both alloys exhibit a similar trend, initially increasing and then decreasing. The increase in conductivity is attributed to the alignment of DPs along the tensile direction, transitioning from a random orientation, which reduces electron scattering and enhances conductivity. However, a high density of dislocations may hinder electron transport after severe deformation, leading to a decrease in conductivity. The increase in conductivity of the fast DP alloy with strain is less than that of the full DP alloy, and it exhibits an earlier decreasing trend. This is due to the overall volume fraction of DPs in the fast DP alloy being lower than that in the full DP alloy, with some solute atoms not fully precipitated. From Figure 3b, it can be seen that the strength of the fast DP alloy is higher than that of the full DP alloy for any amount of deformation, especially after the area is reduced by 97.5% (1070 and 930 MPa for the fast and full DP alloys, respectively).

**Figure 4:**
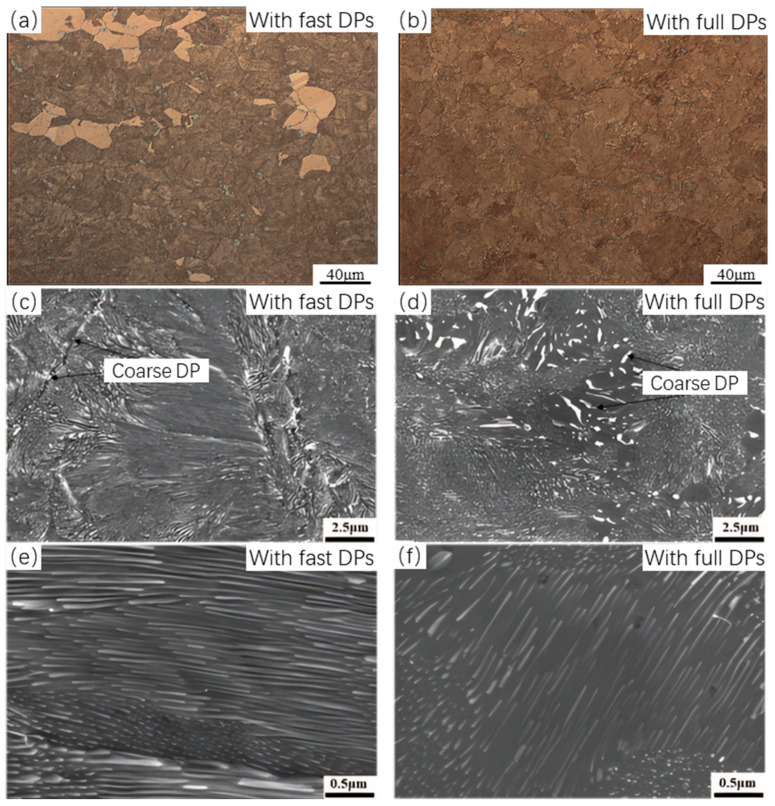
Microstructure of Cu-6Ni-1.42Si alloy with (**a**) fast DPs and (**b**) full DPs; grain boundary morphology (**c**,**d**); precipitate characteristics (**e**,**f**).

Figure 4 shows the microstructure of Cu-Ni-Si alloys after aging, where (a) represents fast DPs and (b) represents full DPs. In the OM, a significant difference in the morphology around the grain boundaries can be observed. The fast DP alloy has narrower grain boundaries with some relatively coarse inclusions distributed near the boundaries, while the full DP alloy exhibits a pronounced coarsening phenomenon at the grain boundaries. Figure 4c,d display scanning electron microscope (SEM) images of the grain boundary morphology. In the fast DP alloy, inclusions with sizes in the order of hundreds of nanometers are found at the grain boundaries, whereas in the full DP alloy, a large number of coarse precipitate phases are observed near the grain boundaries, with sizes of several micrometers. These thicker precipitates are considered to be coarsened DPs formed during prolonged aging. This coarsening occurs because the initially precipitated DPs are finer and have a larger interfacial area, leading to a higher overall interfacial energy in the system. As aging progresses, the system tends to minimize its total energy by reducing the interfacial area, which it achieves by reducing the number of precipitates and increasing their size. During this process, smaller precipitates dissolve through grain boundary migration and diffusion, while some DPs coarsen and gradually form thicker lamellar structures [40].

**Figure 5:**
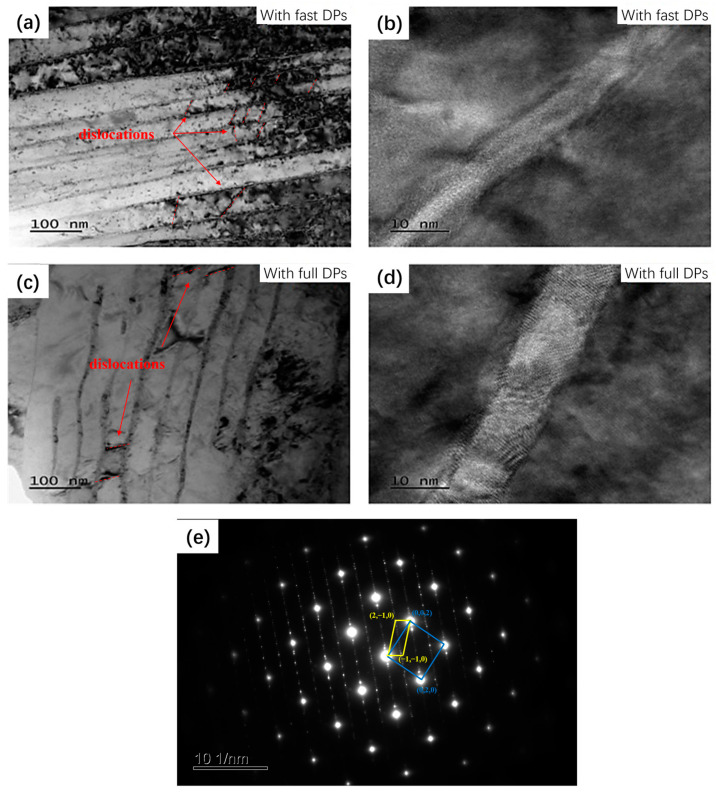
The bright-field TEM images (**a**) and HR-TEM images (**b**) of the fast DP alloy. The bright-field TEM images (**c**) and HR-TEM images (**d**) of the full DP alloy. SADP (**e**) corresponding to (**a**).

Figure 4e,f illustrate that the density of discontinuous phases within the alloy aged for 30 min is higher than that in the alloy aged for 720 min. This indicates that with an increase in aging time, the Ni and Si atoms dissolved in the matrix do not continue to precipitate as finer discontinuous phases; instead, they precipitate as coarse DPs near the grain boundaries [40]. This phenomenon results in a higher volume fraction of fine discontinuous phases within the grains of the fast DP alloy compared to the full DP alloy, thereby exhibiting greater strength. The figures also show that the inter-distance between DPs in the fast DP alloy is smaller than that in the full DP alloy. Measurements of the DP spacing in Figure 5a,c indicate that the average DP spacing in the fast DP alloy is 38.8 nm, while in the full DP alloy, it is 54.5 nm. This smaller spacing contributes more significantly to the overall strength. It can be reasonably hypothesized that during the initial stage of aging, a high precipitate driving force and limited aging time promote the formation of a large number of DPs without coarsening. However, as the aging time extends, both the inter-distance between DPs and the size of the precipitates increases.

From the TEM images in Figure 5, it can also be observed that there are certain differences in the morphology of the discontinuous precipitates between the two alloys. The crystallographic OR between the matrix and precipitates is determined as follows: (0,2,2)_Cu_//(1,1,0)_Ni2Si_, (0,4,2)_Cu_//(2,2,0)_Ni2Si_, [100]_Cu_//[010]_Ni2Si_. The inter-distance between precipitates in the fast DP alloy is significantly smaller than that in the full DP alloy, as shown in Figure 5a,c. In traditional strengthening mechanisms, a smaller inter-distance can contribute to a higher strength in the alloy. Figure 5b,d present HR-TEM images, which reveal that the discontinuous precipitates in the fast DP alloy have smaller thicknesses. Calculations show that the average size of DPs in the fast DP alloy is 5.78 nm, while in the fully developed DP alloy, it is 9.47 nm. The smaller size of DP thickness further contributes to the alloy’s strength. Additionally, it can be observed that in the fast DP alloy, there is a high density of dislocations present between the precipitates, which is greater than that in the full DP alloy. This may be attributed to the long aging time required to achieve full DPs, which leads to the recovery of dislocations generated during pre-deformation, resulting in a reduced dislocation density. Consequently, the work hardening effect caused by dislocation accumulation is also diminished, resulting in the strength of the full DP alloy being lower than that of the fast DP alloy.

**Figure 6:**
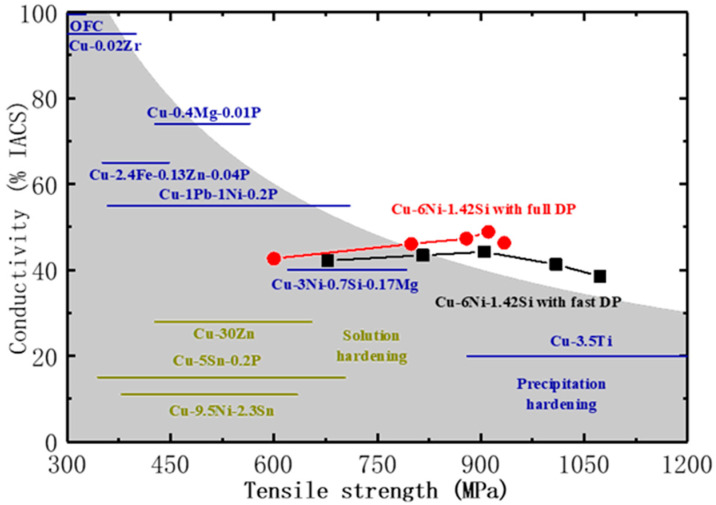
Tensile strength and conductivity of pre-deformed Cu-Ni-Si alloys with fast and full DPs together with previous data reported in Ref. [35].

Figure 6 illustrates the strength/conductivity of pre-deformed Cu-Ni-Si alloys with fast DPs and full DPs under aging and tensile conditions, compared to the performance of traditional solid solution-hardened and precipitation-hardened alloys. The studied alloy demonstrates a superior combination of high strength (1070 MPa) and reasonable conductivity (38.5% IACS) among other reported copper alloys [35]. To further enhance both strength and conductivity simultaneously, it is necessary to adjust the mechanical and thermal treatment processes of the alloy, allowing for the precipitation of finer-sized and more closely spaced DPs that are fully precipitated and uniformly distributed. This will be discussed in future studies.

## 4. Conclusions

This work studied the mechanical properties, and microstructure evolution of Cu–6Ni-1.42Si alloys with pre-deformation. The results are summarized as follows:

1. The formation of DPs was accelerated by pre-deformation before aging, enabling the successful production of fast DP and full DP specimens. The aging time required for complete DP generation was reduced from 24 h to 30 min, representing a 48-fold reduction compared to previous studies [34,35].
2. Compared to traditional Cu-Ni-Si alloys, the fast DP alloy exhibits a higher strength and a good conductivity, with values of 1070 MPa and 38.5% IACS, while the full DP alloy demonstrates a relatively high strength and a superior conductivity, at 930 MPa and 46% IACS, respectively.
3. The higher strength of the fast DP alloy is attributed to the shorter inter-distance of DPs and a smaller DP thickness, approximately 38.8 nm and 5.78 nm, respectively. Moreover, fewer coarsened DPs are present near the grain boundaries, as the shorter aging time effectively restrains DP coarsening behavior.

## Data Availability

Data are contained within the article.
